# *Notes from the Field*: Circulating Vaccine-Derived Poliovirus Type 2 Emergences Linked to Novel Oral Poliovirus Vaccine Type 2 Use — Six African Countries, 2021–2023

**DOI:** 10.15585/mmwr.mm7238a4

**Published:** 2023-09-22

**Authors:** Elizabeth Davlantes, Jaume Jorba, Elizabeth Henderson, Kelley Bullard, Mark. A. Deka, Anfumbom Kfutwah, Javier Martin, Maël Bessaud, Lester M. Shulman, Kaija Hawes, Ousmane M. Diop, Ananda S. Bandyopadhyay, Simona Zipursky, Cara C. Burns

**Affiliations:** ^1^Global Immunization Division, Global Health Center, CDC; ^2^Division of Viral Diseases, National Center for Immunization and Respiratory Diseases, CDC; ^3^Geospatial Research, Analysis, and Services Program, Agency for Toxic Substances and Disease Registry, Atlanta, Georgia; ^4^Regional Polio Laboratory Network, World Health Organization Regional Office for Africa, Brazzaville, Republic of the Congo; ^5^Division of Vaccines, Medicines and Healthcare products Regulatory Agency, Potters Bar, United Kingdom; ^6^Institut Pasteur, Université Paris Cité, Virus Sensing and Signaling Unit, Paris, France; ^7^Central Virology Laboratory, Public Health Services, Israel Ministry of Health, Tel Aviv, Israel; ^8^Bill & Melinda Gates Foundation, Polio Team, Seattle, Washington; ^9^World Health Organization, Geneva, Switzerland.

Circulating vaccine-derived poliovirus (cVDPV) outbreaks can occur when oral poliovirus vaccine strains (most often, Sabin monovalent oral poliovirus vaccine type 2 [mOPV2]) undergo prolonged circulation in undervaccinated populations, resulting in genetic reversion to neurovirulence. A novel type 2 oral poliovirus vaccine (nOPV2) has been developed, which has been shown in clinical trials to be less likely than mOPV2 to revert to paralytic variants and to have limited genetic modifications in initial field use (*1*–*4*). Approximately 700 million doses of nOPV2 have been administered worldwide in response to outbreaks of cVDPV type 2 (cVDPV2). cVDPV2 detections originating from nOPV2 use from initial rollout during March 2021–September 7, 2023, are described in this report.

## Investigation and Outcomes

Polio surveillance and laboratory data collected through the Global Polio Eradication Initiative were reviewed. During August 2021–July 2023, seven cVDPV2 emergences of nOPV2 origin from 61 paralytic cases and 39 environmental surveillance (sewage) samples were detected in six countries, all in Africa: Burundi, Central African Republic (CAR), Democratic Republic of the Congo (DRC), Nigeria, Tanzania, and Zambia (Figure). The isolates have limited divergence from the parental nOPV2 vaccine strain in the VP1 capsid protein coding area (six to 16 nucleotide substitutions), indicating that surveillance detected emergence relatively early after vaccination. This activity was reviewed by CDC, deemed not research, and was conducted consistent with applicable federal law and CDC policy.*

**FIGURE:**
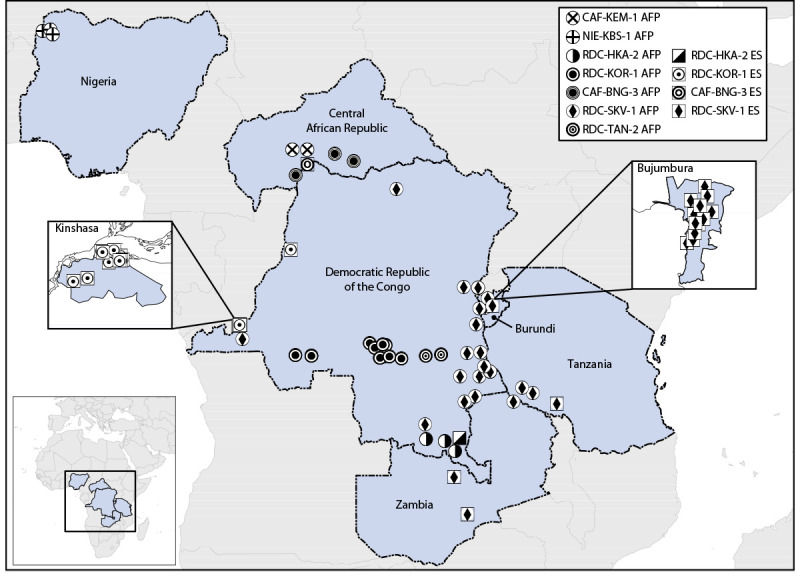
Detections of circulating vaccine-derived poliovirus type 2 linked to novel oral poliovirus type 2 vaccine use, by emergence group — Africa, 2021–2023 **Abbreviations:** AFP = acute flaccid paralysis case; ES = environmental surveillance isolate.

Circulation of four of the seven cVDPV2 emergences derived from nOPV2 use has been detected only in subnational areas of the countries in which they originated (CAR, DRC, and Nigeria), two have spread to other areas of the originating country (CAR and DRC), and one has spread from the originating country (DRC) to neighboring countries (Burundi, Tanzania, and Zambia). Three emergences spreading within or outside of the originating country (all from DRC) might be ongoing, with most recent detections in July 2023. The largest and widest-spreading emergence, RDC-SKV-1, was first detected in DRC’s South Kivu province in September 2022; viruses from this emergence have been identified in six provinces of DRC and in neighboring Burundi, Tanzania, and Zambia. RDC-SKV-1 has been detected as recently as July 2023 in Tanzania. 

## Preliminary Conclusions and Actions

The potential for mutation and reversion to neurovirulence is a rare but recognized risk for all live attenuated oral poliovirus vaccines; extensive use of nOPV2 worldwide since March 2021 suggests that reversion occurs less frequently than with mOPV2 (*4*). A preliminary estimate suggests that cVDPV2 emergences occur after mOPV2 use at a rate of one emergence per 10 million mOPV2 doses administered; for nOPV2, this rate is approximately 10 times lower, at one emergence per 100 million doses. As with all cVDPV emergences, cVDPV2 outbreaks of nOPV2 origin are more likely to occur when nOPV2 supplementary immunization activities (SIAs) do not achieve high coverage in populations with persistently low immunity against polioviruses (*5*). To combat all cVDPV2 outbreaks and reduce future emergences, responding with prompt SIAs that reach all targeted children is essential.

## Footnotes

* 45 C.F.R. part 46.102(l)(2), 21 C.F.R. part 56; 42 U.S.C. Sect. 241(d); 5 U.S.C. Sect. 552a; 44 U.S.C. Sect. 3501 et seq.
